# Reference Values of Flexion and Supination in the Elbow Joint of a Cohort without Shoulder Pathologies

**DOI:** 10.1155/2017/1654796

**Published:** 2017-10-24

**Authors:** Mehmet F. Güleçyüz, Matthias F. Pietschmann, Stefan Michalski, Ferdinand M. Eberhard, Alexander Crispin, Christian Schröder, Maximilian J. Mittermüller, Peter E. Müller

**Affiliations:** ^1^Department of Orthopaedics, Physical Medicine and Rehabilitation, Medical Center of the University of Munich (LMU), Munich, Germany; ^2^Department of Traumatology and Orthopaedics, Helios Klinik München Perlach, Munich, Germany; ^3^Department of Urology, Diakonie-Klinikum Stuttgart, Stuttgart, Germany; ^4^Institute for Medical Information Processing, Biometry and Epidemiology, Medical Center of the University of Munich (LMU), Munich, Germany; ^5^Laboratory of Biomechanics and Experimental Orthopaedics, Medical Center of the University of Munich (LMU), Munich, Germany; ^6^Munich University of Applied Sciences, Munich, Germany

## Abstract

**Background:**

After surgery of the long head of the biceps tendon, the examination of the biceps brachii muscle function and strength is common clinical practice. The muscle strength is usually compared with the uninjured contralateral side or with a matched pair group assuming that the uninjured side can be used as an appropriate reference.

**Hypothesis/Purpose:**

The purpose of this study was to define reference values of the supination and flexion strength in the elbow joint and to investigate the influence of the arm positions and various anthropometric factors.

**Methods:**

105 participants without any shoulder pathologies were enrolled. A full medical history was obtained and a physical examination was performed. The bilateral isometric testing included the supination torque in various forearm positions and elbow flexion strength with a custom engineered dynamometer. Multiple linear regression analysis was used to investigate the correlation of the strength and anthropometric factors.

**Results:**

Only age and gender were significant supination and flexion strength predictors of the elbow. Hence, it was possible to calculate a gender-specific regression line for each forearm position to predict the age-dependent supination torque. The supination strength was greatest with the arm in 90° elbow flexion and the upper arm in full pronation.

## 1. Introduction

The main function of the biceps brachii muscle and its proximal tendons in the shoulder is stabilization, assistance in arm abduction, and flexion and internal rotation. In the elbow joint, it acts as a flexor and as a supinator, especially at 90° elbow flexion [1–3]. Forearm supination is possible with the interaction of the supinator muscle and the biceps brachii muscle, where the biceps brachii muscle mainly contributes to the strength.

Lesions of the long head of the biceps tendon (LHBT) are a common cause of pain and functional impairment of the shoulder [4–7]. After surgical LHBT treatment, the examination of the biceps brachii muscle function and strength is common clinical practice. The strength of the biceps brachii muscle is usually compared with the uninjured contralateral side or with a matched pair group, assuming that the uninjured side can be used as an appropriate reference [8].

Both the LHBT tenotomy and the tenodesis present comparable postoperative clinical examination results, regardless of the cosmetic outcome, that is, potential risk of muscle belly distalization in terms of “Popeye's sign.” Generally, postoperative clinical examinations assess the gross manual muscle function and therefore lack objectivity and reliability. Hence, it is difficult to recommend one procedure over the other, especially if the quantification of the potential deficit of the biceps brachii muscle function cannot be performed [8–11]. Review of the current literature shows that neither reference values nor the impact of anthropometric factors affecting the strength of the elbow supination and flexion exists [12].

The aim of this study is to investigate the supination and flexion strength of the elbow joint—the main function of the biceps brachii muscle—from a large cohort of healthy participants without a history of LHBT injury. The secondary aim is to analyze possible anthropometric factors of influence. We hypothesize that it is possible to define predictive values for each individual of any given age with multiple linear regression analysis.

## 2. Materials and Methods

### 2.1. The Participants

A total of 105 participants (55 men, mean age: 50.6 ± 21.9 years; 50 women, mean age: 53.6 ± 21.7 years) without any shoulder pathologies, especially of the LHBT, were enrolled. This study received approval from the Local Research Ethical Committee. Power analysis was performed prior to testing in cooperation with the Institute for Medical Information Processing, Biometry and Epidemiology of our university. Participants with no history of shoulder disorders were acquired in our outpatient clinic and asked to participate in this study. In the younger and older age cohort, individuals were systematically addressed on campus and in nursing homes for the elderly.

The following criteria for exclusion were applied: impaired state of health or general condition; reduced range of motion of the upper extremities (shoulder, elbow, and hand); positive Jobe, Starter, Speed, Palm-Up, or Yergason's sign; pain in the upper extremity during testing or rest; a history of a traumatic event; and a neurological or inflammatory disease.

The following anthropometric factors were assessed: gender, handedness, age (in years), occupation, and athletic activity (including frequency and level of activity). In addition, the following values were measured: height; weight; body mass index; bilateral length of the upper and lower arm; bilateral circumference of the upper arm, the wrist, and the metacarpus; and skinfold measurement of the dorsal and ventral upper arm. The height, weight, and body mass index (BMI) were compared to the average values in the German population as listed on the homepage of the Federal Statistical Office [13]. The percentage of body fat was determined with a skinfold and compared to the age- and gender-dependent tables by Donoghue [14].

### 2.2. Strength Measurement

**Figure 1:**
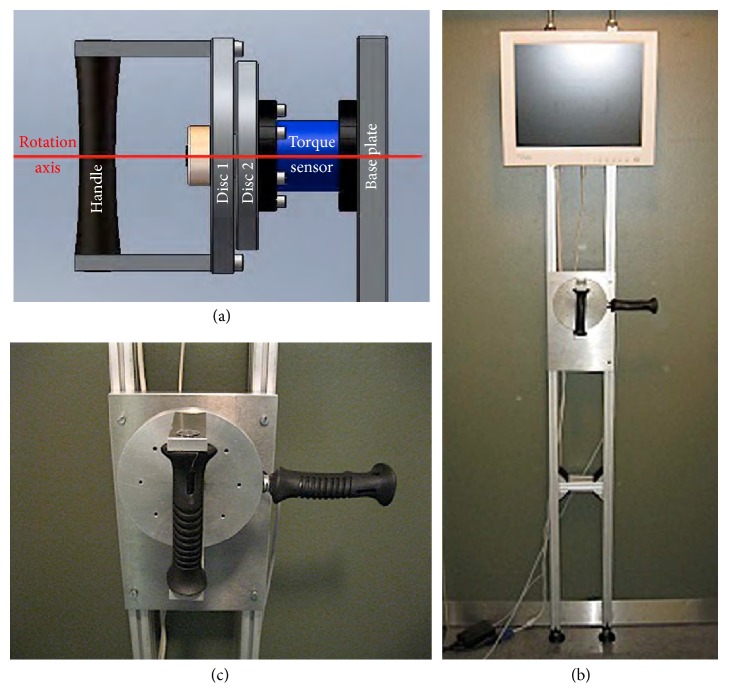
(a) Schematic design of the custom dynamometer with the vertical ergonomic handle bar, disc 1, disc 2, torque sensor, and base plate (from left to right). (b) The tracks allow subject-dependent adaptation of the height of the dynamometer. The dynamometer is connected to the computer. (c) Details of the two ergonomic handle bars. The vertical handle bar is intended for the supination strength whereas the horizontal one is intended for the flexion strength measurement.

The isometric strength tests were performed with a custom engineered dynamometer consisting of two rotating aluminum discs interlocking at defined angles. Two ergonomic handle bars are attached to the outside disc facing the subject: one vertically on the front face and the other horizontal on the right side of the outside disc for measuring the supination and flexion strengths, respectively. The second disc, away from the subject, is affixed to a nonrotating torque sensor (Type 8627 Burster Präzisionsmesstechnik GmbH & Co. KG, Gernsbach, Germany) and the base plate; the sensor has a linearity error of 0.1% of the full scale (FS). The base plate is mobile on vertical tracks and can be adjusted to the height of the subject. The moment of supination and flexion with the torque sensor and A/D-transformer is analyzed with a computer and the graphical computer language software Laboratory Virtual Instrument Engineering Workbench (LabVIEW) (National Instruments Corporation, Austin, TX, USA). The custom engineered dynamometer is visualized in Figure 1.

**Figure 2:**
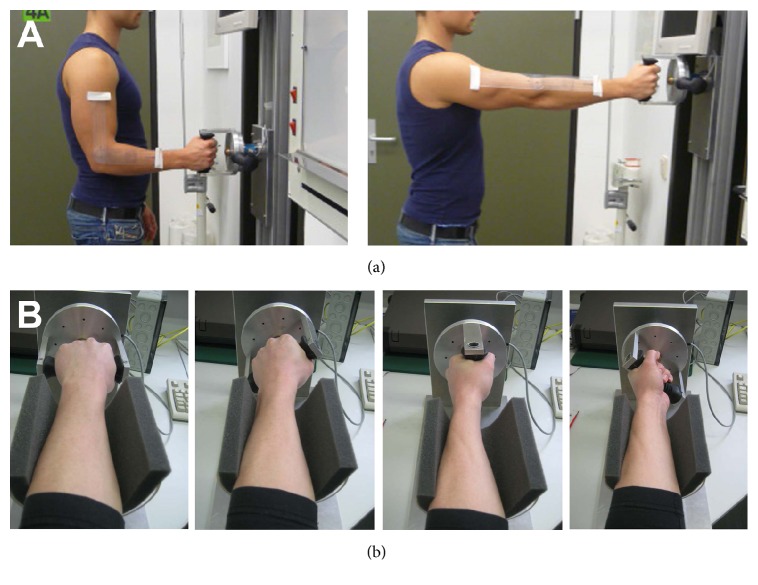
(a) Position of the elbow at 90° flexion and at 0° full extension. The positions were verified with a goniometer. (b) The four different forearm starting positions of the supination strength measurement at 0°, 45°, 90°, and 135° supination angles (from left to right) for the left arm.

The testing was performed in a randomized manner and every participant underwent all measurements. The participants were introduced to the dynamometer by a single examiner to get accustomed with the device. A green signal on the computer monitor in front of the subject initiated the beginning of the isometric strength measurement and was stopped at a red signal. Each measurement was performed three times per position and the mean value was calculated. Both upper limbs were tested in an alternating manner. The testing was performed at intervals of three minutes between measurements to allow recuperation of the muscle groups. The supination strength in newton meters (Nm) was tested in two elbow positions. In position 1, the shoulder is in a neutral rotation, adducted to the body, and the elbow is flexed at 90° (Figure 2). This represents the position in which the biceps brachii muscle has the maximal impact on supination strength. In position 2, the shoulder is in 90° anteversion and the elbow is fully extended at 0°. This is the position where the biceps brachii muscle has the lowest impact on supination strength.

Furthermore, the supination strength was tested in four different starting positions and supination intervals of 45° starting from 90° pronation of the forearm: 0° supination, 45° supination, 90° supination, and 135° supination (Figure 2). This resulted in a total of 8 different positions of the arm and therefore 8 tests per subject in a randomized manner. Position “90_0,” for example, means that the elbow was flexed at 90° and the forearm was held in 0° supination. Position “0_90” on the other hand means that the elbow was fully extended and the forearm was supinated at 90°. All the possible elbow and forearm positions are summarized in Table 1.

### 2.3. Data Analysis and Statistics

Seven age groups with seven participants representing the decades of the adult population were enclosed in this study (see Table 2). The higher number of young male participants is due to the model validation and to the power analysis prior to testing.

Statistical data analysis was performed with SAS (Statistical Analysis System) software, Version 9.2 (SAS Institute Inc., Cary, NC, USA), and GraphPad Prism software, Version 6 (GraphPad Software Inc., La Jolla, CA, USA). The paired *t*-test was used for the comparison for dependent samples and the Mann–Whitney *U* test was used for independent samples. Multiple independent samples were analyzed with the Kruskal-Wallis test and Dunn's post hoc test. Statistical significance was calculated based on a 5% level (*p* < 0.05) and marked in the diagrams with an asterisk: ^*∗*^*p* ≤ 0.05, ^*∗∗*^*p* ≤ 0.01, and ^*∗∗∗*^*p* ≤ 0.001. Data was visualized with bar diagrams.

With the multiple linear regression analysis including a forward selection, the anthropometric variables were correlated to the measured supination/flexion strength of the forearm positions. Forward selection was based on *F* tests for the regression coefficients. Independent variables with *p* values less than 0.05 were included in the regression models; significance was also calculated based on a 5% level (*p* < 0.05). The multicolinearity and anthropometric variables problem was reduced by using the Principal Component Analysis. A matrix of rotating factors was calculated with the varimax rotation with the SAS software. An original variable was determined for all rotating components with the highest factor and was included in the multiple linear regression model—dependent on the *p* values—and was defined as the leading variable. The dominant arms of the participants were tested consecutively in the 8 possible arm positions, and then the contralateral nondominant arms were tested.

## 3. Results

### 3.1. Anthropometric Data

A total of 53 male participants (96.4%) and 45 female participants (90%) were right-hand dominant. The mean height, weight, and body mass index of the male and female participants were comparable to the average values of the German population measurements of the Federal Statistical Office [13]. The mean skinfold measurement of the dorsal and ventral upper arm of the male and female participants was higher than the average German population and measured 25.6 ± 7.1% and 32.8 ± 5.4%, respectively [13]. There were no differences between the male and female participants or between the right and left side regarding the length of the upper and lower arm and the bilateral circumference of the upper arm, the wrist, and the metacarpus.  Table 3 shows a summary of the mean values of the anthropometric data.

### 3.2. Supination Strength

The retrieved mean supination strength and the standard deviations according to the arm positions and age are summarized in Table 4.

**Figure 3:**
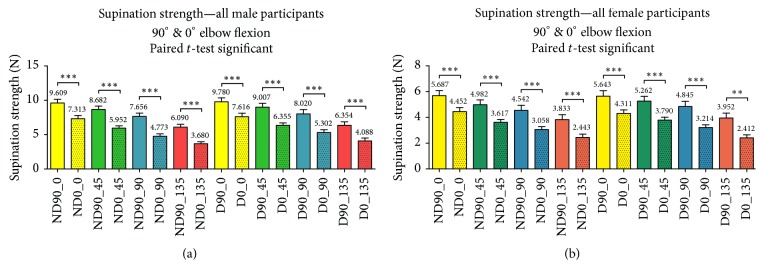
The supination strength of the (a) male and (b) female participants and starting positions of the forearm were compared at 90° elbow flexion and 0° elbow flexion. There was statistical significance between the elbow positions for both sexes.

There was a significant increase (*p* ≤ 0.0001) in mean supination strength at 0°, 45°, 90°, and 135° positions of the forearm at 90° elbow flexion in comparison to full elbow extension at 0° in both male and female participants (see Figure 3).

Participants below 39 years of age showed significantly higher supination strength using the Wilcoxon test in the dominant arm compared with the nondominant arm. This was predominantly observed in the male subgroup (8 arm positions); the female subgroup showed a significant difference in 1 arm position. The results of the arm dominance are summarized in Table 5. The strength in the dominant arm diminishes with progressing age in both sexes.

### 3.3. Flexion Strength

**Figure 4:**
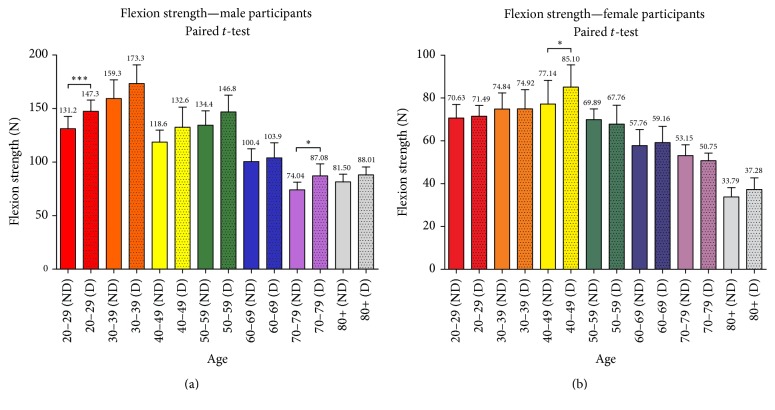
Flexion strength in male (a) and female (b) participants in the different age groups. The flexion strength decreases with progressing age.

Male participants reached their maximum flexion strength, independent of the arm dominance in the 4th decade (30–39 years); with progressing age, the flexion strength in male participants decreases. Significance in arm dominance was visible only in the third decade with *p* ≤ 0.001 and in the eighth decade (70–80 years) with *p* ≤ 0.05. The female participants reached their maximum flexion strength in the 5th decade (40–49 years). Arm dominance was only visible in the 5th decade with *p* < 0.05. The flexion strength of the male population was significantly higher than the female population with *p* < 0.0001. The data regarding the flexion strength for both sexes is visualized in Figure 4.

### 3.4. Regression Analysis of Anthropometric Factors

The forward selection of anthropometric factors showed that only the age and gender of the assessed participants were significant predictors (*p* < 0.05) to the supination strength of the 8 different forearm positions and arm dominance. All other anthropometric factors did not show an effect on the supination strength.

The age and gender had a significant predicting effect on the flexion strength of the dominant arm with *p* < 0.05. The upper arm length showed an additional predicting effect on the flexion strength of the nondominant arm (*p* < 0.05). The other anthropometric factors did not influence the flexion strength.

**Figure 5:**
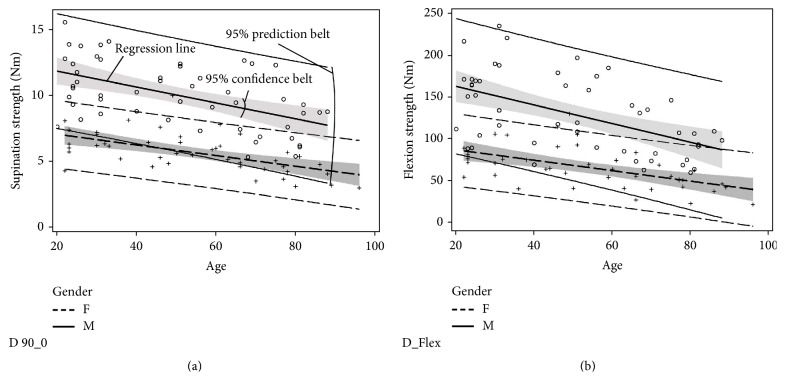
The age and gender of the participants are the only significant predictors of the supination strength (a) and flexion strength (b) in the regression analysis of the dominant arm.

Figure 5 shows an example of the graphical visualization for the dominant arm at the 90_0 position (90° elbow flexion and 0° supination).

## 4. Discussion

This study generated gender specific reference values for the supination and flexion strength of the elbow joint for LHBT healthy adults aged 20 and above. Anthropometric factor analysis showed that only age and gender of the cohorts were significant predictors of the supination and flexion strength.

Although several studies exist in testing the strength of the forearm, this study is the first to analyze the influence of the elbow angle and supination angle on the torque and to develop an age related baseline. This baseline measurement can be used as a reference for comparison purposes in future studies.

O'Sullivan and Gallwey investigated the forearm torque at different angles of the upper extremity and the muscular activity via an electromyography of the main torque muscles [15]. The maximum supination strength was recorded with an increasing flexion of the elbow and pronation of the forearm. The greatest measured effect of the biceps brachii on the supination was seen with the elbow flexed at 90° and the forearm in neutral or slightly supinated forearm position. In contrast, the lowest impact was registered with a maximal extension in the elbow and a pronated forearm [15].

Winters and Kleweno analyzed the strength of the upper limb with the use of a kinetic communicator (Kin-Com) exercise system (Chattex Corp., Chattanooga, TN, USA) and three-dimensional upper limb model [16]. They observed that the influence of the biceps brachii muscle on the supination strength was minimal with the 90° shoulder flexion and adduction and 0° elbow extension and that the supination strength increases with increasing pronation [16]. The fact that the greatest supination torque is achieved out of a submaximum pronation position of the forearm could be verified in several other studies as well [17–19].

In some study setups, the forearm of the subject lays on an adjustable armrest preventing evasive movements [16, 19]. In our setup, the participants were instructed to keep their upper limb in the designated positions during the motion sequence allowing more natural kinesthesia. The correct motion sequence was nevertheless under the close surveillance of the examiner, positions were corrected via a goniometer, and evasive moments were prevented.

The strength tests were conducted with alternating arms and a resting pause of at least three minutes was maintained to rule out early tiring as implemented in similar setups [15, 16, 18–22].

Günther et al. described a positive correlation between the grip strength and the circumference of the metacarpus, wrist, and upper arm [21]. These variables were included in our study but showed no significant influence on the supination or flexion strength of the arms.

Young male adults between 20 and 39 years of age yielded the highest supination strength at 0° elbow extension and 90° elbow flexion starting from the 0° supination position. This was similar for the female population as well. The supination values for both sexes were comparable to the results yielded in other studies with a similar setup [15, 16, 19]. The observation that increasing elbow flexion results in increased supination strength was also comparable to other studies [15, 16, 23]. This was also independent of the gender of the participants. The starting position of the forearm showed a decrease of the supination strength with increasing supination position (Table 5) [15–19, 24, 25]. This was significant for the male and female participants and the nondominant and dominant arm from the 90° supination position and 90° elbow flexion. The generated torque during forearm rotation is dependent on the position of the pronators and supinators. The supinator and biceps brachii muscles can develop torque 4 times greater if the supination is initiated in a pronation position [25].

The flexion strength of the males was significantly higher than that of the female group. The values yielded in this study were higher than the values in other experimental setups [16, 24]. The only difference in our setup in comparison to the others was that our participants were standing rather than sitting. Whether the standing or sitting position affects the generated flexion torque remains unclear and requires further investigation. We observed significance in arm dominance only for the male group in the 3rd and 8th decades and for the female group in the 5th decade. This indicates that, in all other age decades, the contralateral limb can be used as a good reference by the clinician. Shank et al.'s statement that the dominant arm results in a significantly higher flexion strength could not be proven, especially when considering the fact that they observed 21 male and 10 female participants [24].

The forward selection of anthropometric variables and multiple linear regression analysis with the supination and flexion strength resulted in the development of a novel prognostic, age- and gender-dependent baseline reference. The forward selection of anthropometric variables and multiple linear regression analysis with the supination and flexion strength resulted in the development of a novel prognostic, age- and gender-dependent baseline reference with high reliability of the predictive values.

### 4.1. Limitations

The participants were recruited in the metropolitan area of our city with additional participants from the rural areas and residents of homes for the aged. Thereby, the urban and suburban population is represented.

The study was restricted to Europe typical Caucasian population. In existing publications, a greater muscle strength was described for the African population [15].

The forearms of our participants were not positioned in an adjustable armrest to prevent evasive movements during supination strength testing and the participants were standing rather than sitting. It remains unclear whether these factors could affect the generated torque and requires further investigation.

## 5. Conclusion

This study was designed to define reference values of supination and flexion strength of the forearm in various elbow and forearm positions as a baseline reference in a healthy population with a large sample size in an adult Caucasian population subgrouped in decades. Furthermore, the influence of multiple anthropometric factors was investigated.

The multiple linear regression analysis shows that only the age and the gender have a significant predictive impact on the supination and flexion strength. Other anthropometric factors did not have or had only a very minor influence.

The data from this study could be used as a reference for comparing the healthy population to a LHBT impaired or injured population of a specific gender or age group.

## Figures and Tables

**Table 1:** The elbow position at 90° flexion and 0° extension in comparison to the forearm position results in a total of 8 different initial test positions.

Abbreviation	90_0	90_45	90_90	90_135	0_0	0_45	0_90	0_135
Elbow flexion	90°	90°	90°	90°	0°	0°	0°	0°
Forearm position in relation to 90° pronation	0°supination	45°supination	90°supination	135°supination	0°supination	45°supination	90°supination	135°supination

**Table 2:** A total of 105 participants were included in this study.

Age (years)	Male	Female	Total
20–29	13	8	21
30–39	7	7	14
40–49	7	7	14
50–59	7	7	14
60–69	7	7	14
70–79	7	7	14
80+	7	7	14

Total	55	50	105

**Table 3:** Summary of the mean values of the various anthropometric variables of the examined male and female healthy participants.

	Male	Female
Age	50.6 ± 21.9	53.6 ± 21.7
Right-handed	96.4%	90%
Left-handed	3.6%	10%
Height (cm)	178.98 ± 6.23	164.14 ± 7.46
Weight (kg)	83.22 ± 12.86	65.06 ± 12.70
BMI	25.90	24.10
Length upper arm right (cm)	29.24 ± 1.91	26.96 ± 1.95
Length upper arm left (cm)	29.1 ± 1.73	26.82 ± 1.98
Length underarm right (cm)	27.81 ± 1.97	24.54 ± 0.94
Length underarm left (cm)	27.63 ± 1.94	24.34 ± 0.95
Circumference upper arm right (cm)	29.16 ± 2.74	26.78 ± 3.20
Circumference upper arm left (cm)	28.78 ± 2.76	26.47 ± 3.22
Circumference wrist right (cm)	17.25 ± 1.17	15.58 ± 1.62
Circumference wrist left (cm)	17.24 ± 1.17	15.59 ± 1.64
Circumference metacarpal right (cm)	21.79 ± 1.18	19.09 ± 0.81
Circumference metacarpal left (cm)	21.71 ± 1.24	18.97 ± 0.83
Skinfold ventral right (cm)	5.97 ± 2.99	9.53 ± 4.48
Skinfold ventral left (cm)	5.94 ± 2.99	9.59 ± 4.30
Skinfold dorsal right (cm)	11.24 ± 4.93	18.28 ± 6.87
Skinfold dorsal left (cm)	11.27 ± 5.08	17.28 ± 6.00
Body fat (%)	25.6 ± 7.10	32.8 ± 5.40

**Table 4:** The mean supination strength values in newton meters (Nm) for the nondominant (ND) and dominant (D) limbs are matched to the 8 different arm positions.

Arm position/age	20–29	30–39	40–49	50–59	60–69	70–79	80+
Male participants							

ND90_0	10.68 ± 2.16	11.60 ± 1.88	9.39 ± 1.43	10.50 ± 1.60	9.44 ± 2.44	8.08 ± 2.27	7.58 ± 1.49
ND0_0	7.38 ± 1.73	9.55 ± 2.15	7.07 ± 1.43	7.40 ± 1.02	7.98 ± 2.58	6.20 ± 2.31	5.63 ± 0.93
ND90_45	10.22 ± 1.89	9.94 ± 1.67	8.52 ± 1.43	9.43 ± 1.62	8.39 ± 1.74	7.38 ± 2.08	6.90 ± 1.57
ND0_45	6.26 ± 1.14	7.41 ± 1.41	5.74 ± 1.10	6.16 ± 0.69	6.33 ± 1.82	4.87 ± 1.54	4.90 ± 0.83
ND90_90	9.32 ± 1.61	9.13 ± 1.04	8.27 ± 2.09	7.49 ± 0.89	6.75 ± 2.28	6.51 ± 1.64	6.12 ± 1.17
ND0_90	5.71 ± 0.72	5.94 ± 1.01	5.21 ± 1.44	4.63 ± 0.83	4.63 ± 0.55	3.70 ± 0.86	3.59 ± 0.63
ND90_135	7.49 ± 0.90	7.37 ± 1.25	6.89 ± 1.11	5.56 ± 1.37	5.36 ± 1.50	4.84 ± 1.01	5.12 ± 1.35
ND0_135	4.71 ± 0.72	4.41 ± 0.80	4.09 ± 1.12	3.51 ± 0.72	3.38 ± 0.46	3.06 ± 0.75	2.59 ± 0.57
D90_0	11.33 ± 2.32	11.70 ± 2.21	9.92 ± 1.18	10.37 ± 1.82	9.31 ± 2.70	8.24 ± 2.10	7.59 ± 1.62
D0_0	8.26 ± 1.71	10.10 ± 2.46	7.34 ± 0.76	7.44 ± 1.23	7.72 ± 3.14	6.42 ± 2.21	6.02 ± 1.37
D90_45	10.90 ± 1.95	10.65 ± 1.42	9.30 ± 1.14	9.47 ± 1.53	7.87 ± 1.48	7.78 ± 2.07	7.09 ± 1.46
D0_45	7.06 ± 1.71	7.76 ± 1.45	6.32 ± 0.90	6.53 ± 1.00	6.57 ± 2.91	5.06 ± 1.67	5.18 ± 1.15
D90_90	10.03 ± 1.33	9.73 ± 0.73	9.32 ± 2.01	7.87 ± 1.09	6.35 ± 1.48	6.81 ± 1.62	6.02 ± 0.83
D0_90	6.30 ± 0.88	6.90 ± 0.94	5.85 ± 1.14	4.98 ± 0.74	4.99 ± 0.68	4.11 ± 0.96	3.99 ± 0.86
D90_135	7.61 ± 0.87	7.99 ± 0.62	7.60 ± 1.48	5.92 ± 1.14	4.55 ± 1.23	5.37 ± 0.98	5.44 ± 1.18
D0_135	4.77 ± 0.75	5.78 ± 1.02	5.01 ± 1.33	3.67 ± 0.76	3.32 ± 0.78	3.25 ± 0.68	2.81 ± 0.73

Female participants							

ND90_0	5.97 ± 0.93	6.60 ± 1.03	6.39 ± 1.91	6.43 ± 0.84	5.97 ± 1.16	4.66 ± 1.14	3.79 ± 1.08
ND0_0	4.43 ± 1.20	5.48 ± 0.86	5.25 ± 1.21	4.73 ± 0.38	4.31 ± 0.85	3.81 ± 0.76	3.15 ± 0.73
ND90_45	5.30 ± 1.09	5.92 ± 1.07	5.70 ± 1.64	5.51 ± 0.87	5.03 ± 1.21	4.34 ± 0.72	3.07 ± 0.67
ND0_45	3.44 ± 0.61	4.33 ± 0.72	4.02 ± 0.92	4.07 ± 0.38	3.74 ± 0.95	3.07 ± 0.65	2.65 ± 0.58
ND90_90	5.06 ± 1.49	5.05 ± 1.13	5.25 ± 1.34	5.33 ± 1.45	4.81 ± 0.98	3.87 ± 0.72	2.44 ± 0.60
ND0_90	3.24 ± 0.81	3.76 ± 0.92	3.45 ± 0.75	3.43 ± 0.40	3.01 ± 0.73	2.43 ± 0.52	2.09 ± 0.80
ND90_135	4.60 ± 1.45	4.36 ± 0.63	4.58 ± 1.30	4.57 ± 0.72	3.84 ± 0.64	2.69 ± 0.09	2.21 ± 0.71
ND0_135	2.78 ± 0.97	3.01 ± 0.70	3.02 ± 0.73	2.85 ± 0.71	2.31 ± 0.44	1.98 ± 0.77	1.15 ± 0.21
D90_0	6.23 ± 1.18	6.61 ± 0.94	6.30 ± 1.92	6.32 ± 0.83	5.95 ± 1.18	4.45 ± 0.76	3.64 ± 1.16
D0_0	4.43 ± 1.05	5.04 ± 0.79	5.06 ± 0.94	4.52 ± 0.81	4.25 ± 1.01	3.90 ± 0.60	2.97 ± 0.78
D90_45	5.69 ± 1.28	6.14 ± 0.96	5.85 ± 1.59	5.64 ± 0.57	5.60 ± 0.96	4.49 ± 0.46	3.41 ± 1.21
D0_45	3.83 ± 0.83	4.23 ± 0.50	4.31 ± 0.79	3.95 ± 0.36	4.07 ± 0.61	3.60 ± 0.74	2.54 ± 0.73
D90_90	5.46 ± 1.25	5.69 ± 1.01	5.67 ± 1.46	5.29 ± 0.70	5.11 ± 0.55	3.82 ± 0.25	2.88 ± 1.05
D0_90	3.63 ± 0.57	3.64 ± 0.80	3.58 ± 1.09	3.31 ± 0.39	3.33 ± 0.53	2.86 ± 0.57	2.14 ± 0.72
D90_135	4.99 ± 1.64	4.26 ± 0.86	4.57 ± 1.76	4.52 ± 0.30	4.15 ± 0.44	2.96 ± 0.41	2.21 ± 1.33
D0_135	2.59 ± 0.61	2.91 ± 0.53	2.97 ± 1.17	2.65 ± 0.32	2.36 ± 0.57	2.26 ± 0.66	1.15 ± 0.60

**Table 5:** The arm positions and dominance of the young male and young female population (≤39) were compared to the 60–70 age groups and resulted in statistical significance especially for the male population.

Arm position	Male	Male	Female	Female
	(≤39)	(60–70)	(≤39)	(60–70)
90_0 D versus 90_0 ND	n.s.	n.s.	n.s.	n.s.
90_45 D versus 90_45 ND	*p* ≤ 0.05	n.s.	n.s.	n.s.
90_90 D versus 90_90 ND	*p* ≤ 0.01	n.s.	*p* ≤ 0.01	n.s.
90_135 D versus 90_135 ND	n.s.	n.s.	n.s.	n.s.
0_0 D versus 0_0 ND	*p* ≤ 0.001	n.s.	n.s.	n.s.
0_45 D versus 0_45 ND	*p* ≤ 0.01	n.s.	n.s.	n.s.
0_90 D versus 0_90 ND	*p* ≤ 0.001	n.s.	n.s.	n.s.
0_135 D versus 0_135 ND	*p* ≤ 0.05	n.s.	n.s.	n.s.

## References

[1] Kapandji I. A. (2009). *Funktionelle Anatomie der Gelenke: Schematisierte und kommentierte Zeichnungen zur menschlichen Biomechanik*.

[2] Schünke M., Schulte E., Schumacher U., Voll M., Wesker K. H. (2007). *Lernatlas der Anatomie. Allgemeine Anatomie und Bewegungssystem*.

[3] Platzer W. (2009). *Taschenatlas Anatomie*.

[4] Youm T., ElAttrache N. S., Tibone J. E., McGarry M. H., Lee T. Q. (2009). The effect of the long head of the biceps on glenohumeral kinematics. *Journal of Shoulder and Elbow Surgery*.

[5] Khazzam M., George M. S., Churchill R. S., Kuhn J. E. (2012). Disorders of the long head of biceps tendon. *Journal of Shoulder and Elbow Surgery*.

[6] Urita A., Funakoshi T., Amano T. (2016). Predictive factors of long head of the biceps tendon disorders-the bicipital groove morphology and subscapularis tendon tear. *Journal of Shoulder and Elbow Surgery*.

[7] Refior H. J., Sowa D. (1995). Long tendon of the biceps brachii: sites of predilection for degenerative lesions. *Journal of Shoulder and Elbow Surgery*.

[8] Hsu A. R., Ghodadra N. S., Provencher C. M. T., Lewis P. B., Bach B. R. (2011). Biceps tenotomy versus tenodesis: a review of clinical outcomes and biomechanical results. *Journal of Shoulder and Elbow Surgery*.

[9] Slenker N. R., Lawson K., Ciccotti M. G., Dodson C. C., Cohen S. B. (2012). Biceps tenotomy versus tenodesis: clinical outcomes. *Arthroscopy - Journal of Arthroscopic and Related Surgery*.

[10] Frost A., Zafar M. S., Maffulli N. (2009). Tenotomy versus tenodesis in the management of pathologic lesions of the tendon of the long head of the biceps brachii. *The American Journal of Sports Medicine*.

[11] Friedman J. L., FitzPatrick J. L., Rylander L. S., Bennett C., Vidal A. F., McCarty E. C. (2015). Biceps tenotomy versus tenodesis in active patients younger than 55 years is there a difference in strength and outcomes?. *Orthopaedic Journal of Sports Medicine*.

[12] Bohannon R. W. (1986). Test-retest reliabilty of hand-held dynamometry during a single session of strength assessment. *Physical Therapy in Sport*.

[13] Körpermaße nach Altersgruppen und Geschlecht, Statistisches Bundesamt, 2013

[14] Donoghue W. C. (2009). *How to Measure Your % Bodyfat: An Instuction Manual for Measuring % Body Fat Using Skinfold Calipers*.

[15] O'Sullivan L. W., Gallwey T. J. (2002). Upper-limb surface electro-myography at maximum supination and pronation torques: the effect of elbow and forearm angle. *Journal of Electromyography & Kinesiology*.

[16] Winters J. M., Kleweno D. G. (1993). Effect of initial upper-limb alignment on muscle contributions to isometric strength curves. *Journal of Biomechanics*.

[17] Bremer A. K., Sennwald G. R., Favre P., Jacob H. A. (2006). Moment arms of forearm rotators. *Clinical Biomechanics*.

[18] Gordon K. D., Pardo R. D., Johnson J. A., King G. J. W., Miller T. A. (2004). Electromyographic activity and strength during maximum isometric pronation and supination efforts in healthy adults. *Journal of Orthopaedic Research*.

[19] Matsuoka J., Berger R. A., Berglund L. J., An K.-N. (2006). An Analysis of Symmetry of Torque Strength of the Forearm Under Resisted Forearm Rotation in Normal Subjects. *Journal of Hand Surgery*.

[20] Boulangé M., Cnockaert J. C., Lensel G., Pertuzon E., Vigreux B. (1979). Muscular fatigue and rate of tension development. *European Journal of Applied Physiology*.

[21] Günther C. M., Bürger A., Rickert M., Crispin A., Schulz C. U. (2008). Grip strength in healthy caucasian adults: reference values. *The Journal of Hand Surgery*.

[22] Kuroda E., Klissouras V., Milsum J. H. (1970). Electrical and metabolic activities and fatigue in human isometric contraction.. *Journal of Applied Physiology*.

[23] Bechtel R., Caldwell G. E. (1994). The influence of task and angle on torque production and muscle activity at the elbow. *Journal of Electromyography & Kinesiology*.

[24] Shank J. R., Singleton S. B., Braun S. (2011). A comparison of forearm supination and elbow flexion strength in patients with long head of the biceps tenotomy or tenodesis. *Arthroscopy - Journal of Arthroscopic and Related Surgery*.

[25] Haugstvedt J.-R., Berger R. A., Berglund L. J. (2001). A mechanical study of the moment-forces of the supinators and pronators of the forearm. *Acta Orthopaedica*.

